# Distinct Response Inhibition Patterns in Obsessive Compulsive Disorder Patients and Pathological Gamblers

**DOI:** 10.3389/fpsyt.2018.00652

**Published:** 2018-12-04

**Authors:** Semion G. Kertzman, Michael Poyurovski, Sarit Faragian, Ronit Weizman, Koby Cohen, Anat Aizer, Abraham Weizman, Pinhas N. Dannon

**Affiliations:** ^1^Psychiatry Division, Beer-Yaakov-Ness Ziona Mental Health Center, Tel Aviv, Israel; ^2^Sackler Faculty of Medicine, Tel Aviv University, Tel Aviv, Israel; ^3^Tirat Carmel Mental Health Center, Israel University, Tirat Carmel, Israel; ^4^Rappaport Faculty of Medicine, Technion Institute of Technology, Haifa, Israel; ^5^Department of Behavioral Science, Ariel University, Ariel, Israel; ^6^Research Unit, Geha Mental Health Center and Felsenstein Medical Research Center, Petah Tikva, Israel

**Keywords:** OCD, pathological gambling, neurocognitive function, response inhibition, impulse control

## Abstract

**Background:** Obsessive-compulsive disorder (OCD) and pathological gambling (PG) are common disorders. The cognitive models of OCD and PG focus on abnormalities in response inhibition. Although, these functions have been studied in different PG and OCD samples, no study has compared the response inhibition in both.

**Methods:** Medication-naïve OCD (*n* = 61) and PG subjects (*n* = 109) and healthy controls (*n* = 131) performed CPT and Go/NoGo tasks.

**Results:** Compared to healthy controls (HC), PG and OCD groups underperformed on speed and exhibited larger time variability on the CPT and Go/NoGo task. Only in OCD patients, a positive correlation between omission errors and response time (RT) was observed in the CPT. At the Go/NoGo task, a negative correlation between false alarms and RT (a fast-errors trade-off) was significant only in the PG group. The HC group had greater sensitivity values (d') than the OCD and PG groups in the Go/NoGo task. The PG group displayed lower d' values and more conservative response criterion in the CPT. In addition, only the OCD group expressed a high switching cost compared to both the PG and HC groups in terms of the RT and d' values.

**Conclusions:** Both the PG and OCD groups demonstrated impaired response inhibition compared to the HC group. On several measures, the OCD and PG groups showed comparable impairments, and in others these were distinct. Thus, it appears that distinct neurocognitive patterns are involved in performance of the CPT and the Go/NoGo tasks among OCD and PG subjects whose cognitive status is currently under intensive investigation.

## Introduction

People that suffer from obsessive-compulsive disorder (OCD) and pathological gambling (PG) are representing an opposite persistent and maladaptive behaviors (1, 2). Stein and Hollander (2) have reported that PG is the impulsive pole and OCD is compulsive pole of an impulsivity-compulsivity continuum (2). The OCD is characterized as excessive self-control, risk aversion, and the avoidance of potential loss or punishments and harmful overestimation (3). By contrast, the PG is associated with diminished self-control, risk-seeking behavior, and insensitivity to punishment and harm minimization (4).

### Compulsivity and Impulsivity as Polar Opposites of a Continuum

Compulsivity and impulsivity have been viewed as fundamentally distinct phenomena that represent a single continuum (2). Compulsivity has been defined as repetitive, ritualistic acts that are carried out to reduce anxiety and are associated with an inability to halt a specific act or thought that provokes severe discomfort (5, 6). Compulsivity, in contrast to impulsivity, does not fall within the range of normal behavior (7).

Impulsivity's was conceptualized as predisposition toward rapid, unplanned responses to stimuli with no attention to potential negative outcome of responses (8) and is associated with diminished ability to resist urges, gaps in regard to delaying gratification, non-reflective decision making, and premature acts driven by the desire to gain pleasure and hypersensitivity to reward (5). Excessive impulsivity can be a core component of substance use disorders, behavioral addictions (such as gambling), and antisocial and borderline personality disorders (7).

Some studies have suggested an overlap between compulsivity and impulsivity expressed as a rise in tension earlier than committing the act and relief after its execution. Although, both compulsions and impulsive behaviors represent a loss of control and may resemble each other regarding potential harm for the subject, they hold different contexts of subjective feeling. If compulsions of OCD are aimed at controlling anxiety or threats, impulsivity in PG is giving in to the urge of a pleasurable activity. But the feelings related to gambling debts and chasing early losses in an attempt to avoid negative results resemble the OCD feelings by compulsions beyond one's control. If a continuum of impulsivity and compulsivity really exists, the place of PG on this continuum is not clear. Phenomenological models of PG have highlighted the shift from the impulsivity pole to the compulsivity pole (9).

### Compulsivity and Impulsivity as Orthogonal Factors

The distinction between OCD and PG is not clear (10, 11). Some OCD patients demonstrate impulsive symptoms (12–16), and some individuals with PG demonstrate compulsive behaviors (1, 9, 17–21). Moreover, Kashyap et al. (22) introduced the “impulsive-compulsive” subtype of OCD, whereby compulsivity and impulsivity, may represent orthogonal factors that each contribute the different weight in various psychological symptoms (23).

### Response Inhibition in OCD and PG

Both compulsive and impulsive behaviors are characterized by the “illusory control of behavior ”(24) related to impairments of “top-down” cognitive control and response inhibition (25).

In OCD, response inhibition is associated with the inability to stop obsessions and compulsions and has been suggested as a candidate phenotype for OCD (10, 11). Research on response inhibition impairments in OCD has revealed inconsistent and heterogeneous results (26, 27). Some researchers have reported differences between OCD and HC samples (28–30). Others have found no differences among OCD individuals compared to HC individuals (31–35). Two recent meta-analyses showed a medium effect size of response inhibition impairments in OCD from 0.49 to 0.55 (26, 36). Symptoms of OCD were not found to be differentially associated with response inhibition impairments as measured by Go/NoGo tasks (37, 38). Omori et al. (39) found significantly higher number of false alarms on a Go/NoGo task in compulsive checking than the compulsive hand washing (39). These studies have varied in potentially important factors such as the age of the subjects and the use of different variants of Go/NoGo tasks and performance measures.

In PG, the inhibitory system is affected by intense motivational drives, as result to release of disinhibited behavior. Inhibition impairments were the key measurement to explain an inability to quit gambling at a casino after losing money (40). Robbins (25) suggested that diminished response inhibition is related to behavioral impulsivity. Subjects with PG show impaired inhibition performance on both time-limited and time-unlimited tasks (41, 42).

### Response Inhibition Model in the Go/NoGo and Continuous Performance Tests (CPT)

The CPT and Go/NoGo tasks are based on a forced-choice paradigm with fast responses to Go signals and withhold responses to NoGo signals administered in uncertainty. The response inhibition model is a cognitive process required for suppressing dominant but inappropriate responses (43). This model is simple enough for administration to populations with even severe psychopathologies. The continuous performance task (CPT) is a “boring” condition in which the small proportion of Go stimuli provides an expectation that the next stimulus could be a NoGo. The CPT requires an ability to maintain a high level of vigilance while waiting for the next trial to begin and, thus, represents a person's ability to successfully detect a rare Go over time and to maintain a state of readiness to respond (43). In contrast, the Go/NoGo task is an “overload” condition in which the proportion of Go stimuli is very high, that required a tendency making a fast response to a Go stimulus while waiting for the next trial. Thus, the Go/NoGo task taps a capacity to withhold a response (to suppress unwanted actions) to a rare NoGo stimulus and assess the ability high-level inhibitory control over motor responses. The CPT captures regulation of approach and avoidance tendencies when the prepotent response is avoidance, whereas the Go/NoGo task captures regulation of these tendencies when the prepotent response is approach.

Together, the Go/NoGo and CPT tasks are two of the most widely used computer-administered tests of impulsive performance in the clinical literature (25). However, there has been little systematic investigation regarding the ability of these tasks to assess compulsivity because compulsivity “is too ambiguous and confusing” (44).

#### Measures of Response Inhibition During Performance on the Go/NoGo and CPT

It is possible that response inhibition impairments may be associated with some measures of performance but not others (45). In the CPT and Go/NoGo tasks, the following measures were used: (1) the number of false alarms (failures to withhold a response to NoGo stimuli); (2) the number of omission errors (no response to Go stimuli within the response window); (3) the slowness of response time (RT); (4) the variability of RT; (5) the speed–accuracy trade-off, as the measure of a response shifting from accuracy to speed, resulting in faster error responses relative to correct responses; (6) “attenuated response inhibition,” a complex measure that involves response inhibition and set shifting (46); this measure is the difference in average RT during a switch from a previous block to a next block for which the Go and NoGo stimuli are reversed. (41, 46). In regard to OCD, two recent meta-analyses showed effect sizes of set shifting/cognitive flexibility tasks from 0.31 to 0.52 (26, 36), while in regard to PG, mixed results were found (9); (7) perceptual sensitivity (the d' index) is the ability of subjects to discriminate a signal (the Go) from noise (the NoGo) irrespective of other parameters that may influence overall performance; greater d′ indicates greater discrimination; 0 indicates performance at a chance (47); and (8) response criterion (the C index) is the individual's strategy used to make the decision to respond. A C score of 0 indicates the absence of a response bias. Positive values reflect bias toward NoGo responses (more correct rejections and omissions) indicating conservative, risk-averse responding. Negative values indicate a bias toward Go responses (more targets and false alarms) representing liberal, risk-taking responding (48).

The alerting condition with a high presentation rate of a Go stimuli elevated arousal as compared with a slow rate of stimuli. Thus, it can be expected that the Go/NoGo condition should cause a more frequent impulsive responses than the CPT. Two competing can be drawn from previous literature. First predicts that condition of high arousal disrupt differentially performance among PG (impulsive) and OCD (compulsive) participants. Second, hypotheses propose that the same performance decrements in both group reject expectation regarding hyper-arousal of impulsive and hypo-arousal of compulsive ends of continuum. Evidence showing differences between two groups in arousal-induced performance decrements can support this hypothesis.

### Hypothesizes

Our primary aim of this research is to compare OCD and PG performance on the CPT and Go/NoGo tasks. Our research sought to examine the eight performance measures that collectively assess inhibition and switching capacities during performance of the tasks by OCD, PG, and healthy control (HC) samples. We hypothesized that OCD and PG individuals have different patterns of response (the C index) and the ability to discriminate a signal from a noise (the d' index) compared to HC in the performance of CPT and Go/NoGo tasks. Moreover, distinct d' and C indexes may serve to identify phenotypes with specific cognitive profiles within the OCD and PG patients' clinical diagnostic entities. Given the continuum-based conceptual model, we assumed that OCD as a disorder of compulsivity would be associated with a higher C index (conservative responding) compared to HC, while PG as, primarily, a disorder of impulsivity would be associated with a lower C index (liberal responding) compared to HC. Based on recent literature, we expected that PG individuals would be more liberal on the response criterion than HC individuals, but that the OCD group would be more rigid/inflexible during the switch from CPT to Go/NoGo and more conservative on the response criterion than the HC group.

A secondary aim is to explore the evidence for the concept of an Impulsive-Compulsive continuum for OCD and PG disorders by contrasting the CPT and Go/NoGo profiles. Our hypotheses were as follows: (1) if PG and OCD are related to a compulsive profile, their performance profiles should be similar and should differ significantly from HC; (2) Patients with OCD will express performance related to the compulsive nature of their disorder; (3) PG subjects should display performance characteristics related to the impulsive nature of the syndrome; and (4) if impulsivity and compulsivity are opposite poles of a single dimension, then impulsivity and compulsivity should be characterized by the inverse profiles of response inhibition performance.

## Methods

### Subjects

We included 109 consecutive medication-naïve patients suffering from PG. All patients were recruited from outpatient clinics. Exclusion criteria were neurological disorders, mental retardation, alcohol, and substance abuse/dependence, major psychiatric disorders, and treatment with any psychiatric medication during the month prior to the screening interview. A senior psychiatrist (PND) administered a semi-structured diagnostic interview that was conducted according to DSM-IV criteria and the South Oaks Gambling Scale (SOGS) (49). Subjects with SOGS scores below five were not included.

Regarding OCD, 61 adult medication-naïve subjects diagnosed with OCD were recruited from two sites located: in Haifa and Rehovot, Ness Ziona and Beer Yaakov Mental Health Centers. Senior psychiatrists (MP, PND) administered a semi-structured diagnostic interview that was conducted according to DSM-IV criteria. Participants who fulfilled the DSM-IV criteria for OCD and who had no other major psychiatric diagnoses and were 18–65 years old were included. All subjects were able to sign and to understand the informed consent.

The control group included 131 healthy volunteers recruited from staff members and medical students by senior psychiatrists (SK and MP). Exclusion criteria for the control group were any current psychiatric disorders and any lifetime DSM-IV axis I psychiatric disorder such as schizophrenia or bipolar disorder; attention deficit/hyperactivity disorder; obsessive-compulsive disorder; or substance use disorder.

All subjects provided a written informed consent after the experimental procedure and the nature of the neurocognitive tests were fully explained to them. This study was fully approved by local IRB committee's and ministry of Health.

Participants completed a screening questionnaire and an interview that covered the following areas: medical history, illicit drug use, family psychiatric history, personal psychiatric history, color blindness, visual, and/or hearing impairments, smoking, literacy, and native language. All subjects were free of psychoactive or pharmacological treatment for at least 28 days prior to the study (with the exception of nicotine and caffeine).

### Neuropsychological Assessment

We used a computerized special task (AnimaScan Ltd, Ashdod, Israel, 2000) based on the inverse contrast model in which frequent NoGo and rare Go stimuli were later contrasted by the opposite presentation of frequent Go and rare NoGo stimuli (41).

#### Neurocognitive Tasks

The computerized version of the task was administered to all participants between 8:00 and 11:00 a.m. Participants with vision limitations were instructed to wear their glasses or contact lenses. An experimenter present in the room when the participant was performing the tasks. The participation in the study for HC was voluntary and without payment. Participants with OCD and PG were motivated for performance because this evaluation was part of clinical assessment. Compensation for participating of the HC in the study was a free charge consultation about their inhibition capacity and professional advice regarding their neurocognitive assessments.

All stimuli displayed in random order centrally on the screen placed at a distance of 60 cm in front of the participant. The Go stimulus was a red square, and the NoGo stimulus was a black square. The stimulus duration was set at 100 ms and the inter-stimulus interval was 2,000 ms. Participants were instructed to press a response button “as fast as possible without making errors” on the Go trials. The participants were asked to react by pressing the red key each time a red square was displayed but to withhold responses to black squares. Examinees responded with a dominant index-finger button press to the Go stimuli using a computer keyboard. Examinees were guided to keep their fingers over the red key in order to be ready to respond. All participants had a practice session with 30 stimuli to familiarize them with the task. In addition, they were given results at the end of the sample session regarding their performance. Subjects were trained so that they achieved a 100% correct performance level.

A total of 300 stimuli were divided into 4 blocks of 75 trials per block. The first and second blocks represented the CPT, and the third and fourth blocks represented the Go/NoGo task. The CPT is a condition with frequent (80%) NoGo stimuli, and the examinees instructed to detect and generate a response to rare Go stimuli (20% frequency). By contrast, the Go/NoGo is a condition with frequent Go (80%) stimuli, and the subjects were instructed to withhold responding to rare NoGo stimuli (20%). The whole task lasted approximately in 10 min, and either CPT or Go/NoGo are completed in 5 min without a pause.

The software automatically removed responses in which the participants either failed to respond within 2,000 ms of a Go signal or made anticipatory responses earlier than 250 ms after a Go signal.

### Statistical Measurements

Data were analyzed using the SPSS software for Windows (v. 21). All analyses used two-tailed levels of significance. Descriptive statistics were calculated with age, educational level (years), and gender. Parametric (analysis of variance) and non-parametric (χ^2^) analyses were performed to compare differences between groups in regard to demographic characteristics. RTs scores were log transformed. To evaluate differences among groups (PG, OCD, and HC) in trial-to-trial performance, repeated measures analysis of variance (ANOVA) with performance measures (RT, variability of RT, numbers of false alarms and omission errors, d' and C indexes) as dependent variables was conducted. In addition, blocks (1, 2, 3, and 4) and condition (“CPT” and “Go/NoGo task”) were analyzed as within-subject variables. Two conditions of the performance were examined: (i) CPT includes 1st and 2nd blocks; (ii) Go/NoGo task includes 3rd and 4th blocks; (iii) a separate analysis was performed for the “switch” from a fast responding to the Go stimuli to ability to withhold a response to the NoGo stimuli (from 2nd to 3rd blocks) as a measure of flexibility of performance. Shift costs were measured by the mean difference in RTs and accuracy between shift and stay trials: a higher shift cost indicated greater difficulty in changing response from one type of responses to another; (iv) Pearson's test was used to assess the correlation between RT and numbers of errors as an indicator of the speed–accuracy trade-off; and (v) for calculating d' and C values, the following algorithms were used: d' = [z(hit rates) – z(false alarms)] and C = −1[z(hit rates) + z(false alarms)], respectively (50).

## Results

### Study Population

Groups differed by gender (χ^2^ = 9.22, *df* = 2, *p* < 0.05). There was a difference in age among the three groups [*F*_(2, 298)_ = 5.68, *p* < 0.05]. OCD patients were younger (*M* = 32.46, *SD* = 10.18) than PG subjects (*M* = 38.76, *SD* = 13.11) [*t*_(168)_ = 3.26, *p* < 0.05]. There were no age differences between the HC (*M* = 35.67, *SD* = 11.62) and OCD groups [*t*_(190)_ = 1.87, *p* = 0.23] or PG [*t*_(238)_ = 1.92, *p* = 0.14]. There was a difference in educational levels [*F*_(2, 298)_ = 9.61, *p* < 0.01]; HC were more educated (*M* = 14.82, *SD* = 3.08) than both OCD (*M* = 13.16, *SD* = 2.53) [*t*_(190)_ = 2.55, *p* < 0.05] and PG (*M* = 13.46, *SD* = 2.86) [*t*_(238)_ = 4.09, *p* < 0.01]. There were no differences between OCD and PG subjects [*t*_(168)_ = 0.74, *p* = 1]. The effect of gender, age, and education are evaluated at task performances, Pearson's correlation matrix was calculated for each factor within each sample. There were no significant correlations between the factors. Thus, differences in gender, age, and educational level were not entered as covariates.

### Response Time

Analysis revealed the main effect of group on RT [*F*_(2, 295)_ = 205.42, *p* < 0.001, η^2^ = 0.41]. Both OCD [*t*_(190)_ = 3.65, *p* < 0.001; Cohen's *d* = 0.2] and PG subjects [*t*_(238)_ = 5.68, *p* < 0.001, Cohen's *d* = 0.33] demonstrated slower RTs than HC subjects. There were no differences between the OCD and PG groups [*t*_(168)_ = 1.40, *p* = 0.5] (see **Table 2**). There was no main effect of group × blocks interaction on RTs [*F*_(6, 885)_ = 1.16, *p* = 0.34, η^2^ = 0.008].

### Variability of Response Time

**Figure 1:**
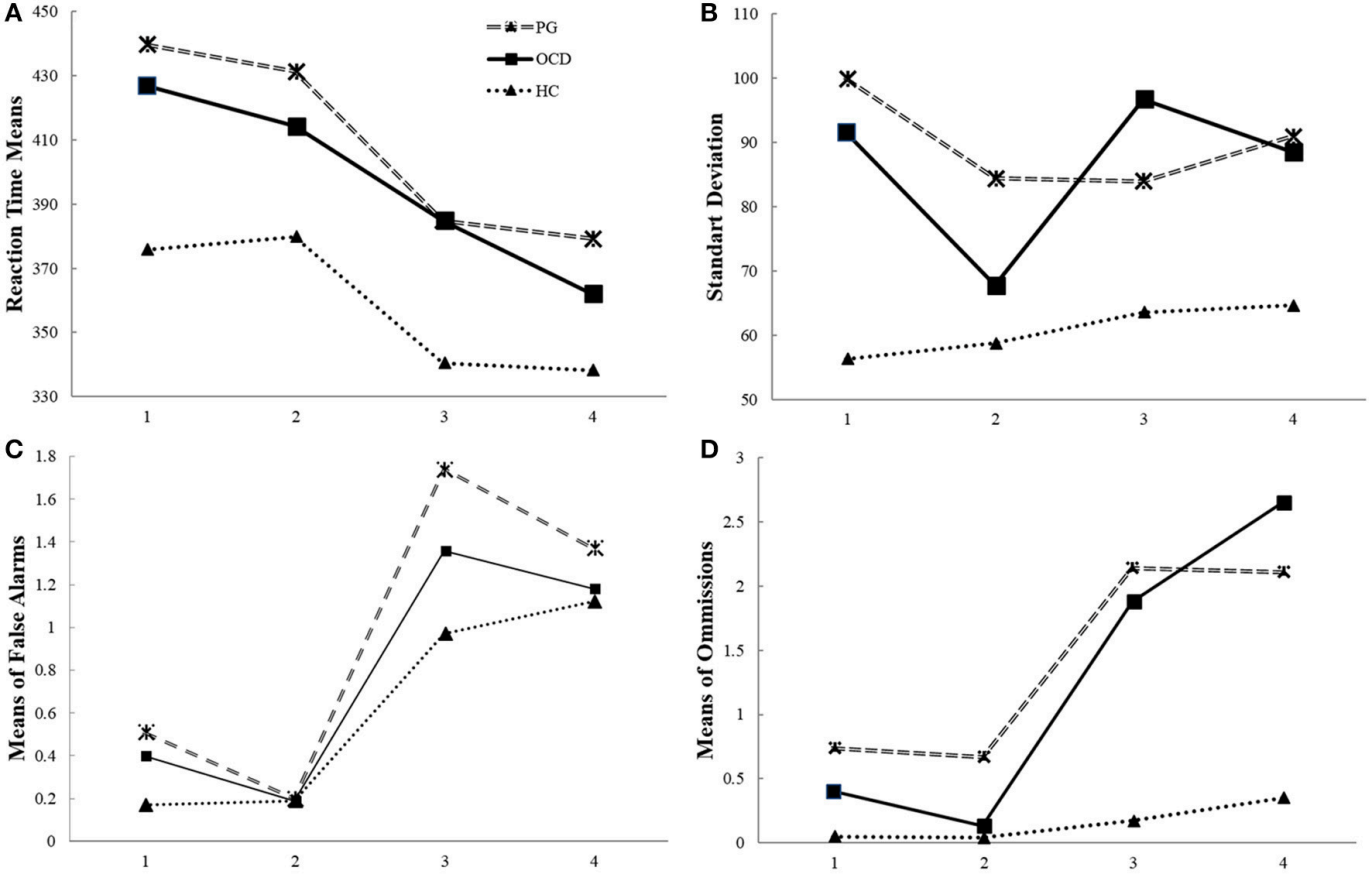
The timeline of manipulation with frequency of Go signal (performance on the CPT and the Go/NoGo conditions of experimental task) in pathological gamblers, obsessive compulsive disorder and healthy control groups. Legend: The CPT (first and second blocks of task) is a “target-detection” condition with frequent NoGo (80%) stimuli, and the subjects were instructed to respond to rare Go stimuli (20%). The randomized, rare Go stimuli promote slow RT and omission errors. The Go/NoGo (third and fourth blocks of task) is an “inhibition” condition with frequent Go (80%) stimuli, and the subjects were instructed to not respond to rare NoGo stimuli (20%).The randomized, frequent Go stimuli that promote fast RT and false alarms. The RT, standard deviation of RT, number of commission and omission errors in the Continuous Performance Test (CPT) and the Go/NoGo task in the pathological gamblers (PG) and obsessive compulsive disorder (OCD) groups compared with the healthy controls (HC). **(A)** Both OCD and PG patients were slower than HC. **(B)** The PG and OCD groups showed more diversity in their performance during both tasks than the control group, as well as in the CPT and the Go/NoGo conditions separately and within blocks; **(C)** There were no differences between the groups in terms of false alarm **(D)** PG patients did more omission errors than the OCD and HC groups in the tasks, there were no differences in terms of omissions between OCD and HC groups.

OCD and PG groups had significantly greater standard deviation of RTs [*F*_(2, 298)_ = 16.32, *p* < 0.01, η^2^ = 0.09] than the HC group. There were main effects of block [*F*_(3, 298)_ = 4.95, *p* < 0.05, η^2^ = 0.04] and a significant interaction of groups and condition [*F*_(6, 894)_ = 3.29, *p* < 0.05; η^2^ = 0.04]. This interaction effect indicates that HC subjects were less diverse in blocks 1, 3, and 4 compared to the OCD patients [*t*_(190)_ = 3.10, *p* < 0.01; *t*_(190)_ = 4.72, *p* < 0.01; *t*_(190)_ = 3.69, *p* < 0.01] and PG [*t*_(238)_ = 5.49, *p* < 0.01; *t*_(238)_ = 4.10, *p* < 0.01; *t*_(238)_ = 4.69, *p* < 0.01] subjects. In block 2, there were no differences in RT variability between the OCD and HC groups [*t*_(190)_ = 1.26, *p* = 0.33]. In addition, OCD subjects had more variability in block 2 compared to blocks 1, 3, and 4 [*t*_(60)_ = 2.26, *p* < 0.05; *t*_(60)_ = 3.15, *p* < 0.01; *t*_(60)_ = 2.80, *p* < 0.01]. This pattern was different for PG subjects, who were more diverse in block 1 than in block 2 and block 3 [*t*_(108)_ = 2, *p* < 0.05; *t*_(108)_ = 2.15, *p* < 0.05]. In addition, for HC subjects, there were no differences in variability levels between the blocks (see Figure 1).

### Errors

Table 1 shows the different errors.

**Table 1:** Adjusted means (and standard deviations) of performance on the CPT and the Go/NoGo task in pathological gamblers, obsessive compulsive disorder and healthy control groups.

**Variable**	**CPT**		**Go/NoGo**	
	**Block 1**	**Block 2**	**Block 3**	**Block 4**
**REACTION TIME**				
PG	2.632 (0.09)	2.621 (0.10)	2.579 (0.10)	2.575 (0.10)
OCD	2.623 (0.07)	2.619 (0.06)	2.574 (0.08)	2.556 (0.09)
HC	2.572 (0.05)	2.575 (0.06)	2.572 (0.06)	2.524 (0.06)
**REACTION TIME VARIABILITY**				
PG	99.93 (81.82)	84.39 (77.78)	83.97 (50.04)	90.93 (57.70)
OCD	91.55 (83.24)	67.80 (45.72)	96.74 (71.87)	88.57 (64.03)
HC	56.35 (35.81)	58.78 (46.40)	63.58 (24.68)	64.63 (25.56)
**FALSE ALARMS**				
PG	0.51 (1.04)	0.20 (0.57)	1.17 (1.62)	1.37 (1.65)
OCD	0.40 (1.14)	0.19 (0.57)	1.36 (2.14)	1.18 (1.51)
HC	0.17 (0.43)	0.19 (0.41)	0.97 (0.96)	1.12 (1.11)
**OMISSION**				
PG	0.74 (1.66)	0.67 (2.29)	2.14 (8.51)	2.11 (8.54)
OCD	0.40 (1.30)	0.13 (0.34)	1.88 (4.60)	2.65 (9.31)
HC	0.05 (0.22)	0.04 (0.24)	0.17 (0.48)	0.35 (0.78)
**d' VALUES**				
PG	3.87 (0.59)	3.97 (0.69)	3.59 (0.80)	3.53 (0.80)
OCD	4.01 (0.52)	4.13 (0.21)	3.52 (0.91)	3.59 (0.91)
HC	4.16 (0.14)	4.14 (0.13)	3.84 (0.35)	3.75 (0.43)
**C VALUES**				
PG	0.32 (0.19)	0.30 (0.14)	−0.37 (0.28)	−0.40 (0.29)
OCD	0.29 (0.12)	0.27 (0.07)	−0.34 (0.27)	−0.34 (0.34)
HC	0.26 (0.06)	0.26 (0.07)	−0.42 (0.17)	−0.42 (0.20)

*RTs presented after log transformation; the d' index is the ability of subjects to discriminate a signal from noise, greater d′ indicates greater discrimination; the C index is the individual's strategy used to make the decision to respond. Positive values reflect bias toward NoGo responses (more correct rejections and omissions) indicating conservative, risk-averse responding. Negative values indicate a bias toward Go responses (more targets and false alarms)*.

#### False Alarms

Analysis failed to reveal any main group effect [*F*_(1, 298)_ = 2.23, *p* = 0.11] or interaction of group × blocks [*F*_(6, 594)_ = 1.21, *p* = 0.29] (see Figure 1C).

#### Missing Responses

Analysis revealed the main effect of group on rates of omission errors [*F*_(1, 298)_ = 4.76, *p* < 0.01, η^2^ = 0.03]; PG subjects made more omission errors than did HC subjects [*t*_(238)_ = 2.85, *p* < 0.01, Cohen's *d* = 0.37]. There were no differences in omission errors in the OCD vs. HC groups [*t*_(168)_ = −0.21, *p* = 1; *t*_(190)_ = 4, *p* = 0.1].

##### Response time during switch from rare go to frequent go stimuli

Results indicated a significant effect of interaction of group × blocks on omission error levels [*F*_(6, 594)_ = 2.68, *p* < 0.05] (see Figure 1D). There were significant differences between PG and HC subjects in the both CPT (blocks 1 [*t*_(238)_ = 4.68, *p* < 0.05], 2 [*t*_(238)_ = 3.14, *p* < 0.01]), and the Go/NoGo task (3 [*t*_(238)_ = 2.64, *p* < 0.01], and 4 **[***t*_(238)_ = 2.34, *p* < 0.05]). There were significant differences between the OCD and HC groups only in the Go/NoGo task (blocks 3 [*t*_(190)_ = 4.2, *p* < 0.01)] and 4 [*t*_(190)_ = 2.8, *p* < 0.05]).

The PG group had more omissions than the OCD group only in block 2 [*t*_(168)_ = 1.85, *p* < 0.05]. If for the HC group, there were no differences in omissions between each block, for the OCD and PG groups, there were significant differences between the CPT and Go/NoGo task: block 1 and block 3 [*t*_(60)_ = 2.47, *p* < 0.05; *t*_(108)_ = 2.03, *p* < 0.05] and block 4 [*t*_(60)_ = 1.87, *p* < 0.05; *t*_(108)_ = 1.91, *p* < 0.01] and in block 2 vs. block 3 [*t*_(60)_ = 3.03, *p* < 0.01; *t*_(108)_ = 2.34, *p* < 0.01] and block 4 [*t*_(60)_ = 2.14, *p* < 0.01; *t*_(108)_ = 2.26, *p* < 0.05].

### Speed–Accuracy Trade-Off

In the CPT, the number of false alarms was negatively correlated with RTs in the HC group, but in the PG group, the subjects' correlation was positive. In OCD group there were no significant correlation between RT and false alarms. In the all groups, there was a positive correlation between RTs and omission errors (see Table 2).

**Table 2:** Correlation between response time and number of errors (false alarms and omissions) in pathological gamblers, obsessive compulsive disorder and healthy control groups.

		**PG**	**OCD**	**None**
CPT	False alarms	**0.21**	0.14	−**0.23**
	Omissions	**0.28**	**0.28**	**0.16**
Go/NoGo	False alarms	−**0.25**	–0.1	−**0.31**
	Omissions	**0.21**	**0.46**	0.05

bolds indicate p < 0.05

In Go/NoGo, a negative correlation between false alarms and RT was significant in the PG and HC groups. A positive correlation between omission errors and RT was observed in OCD and PG patients but in HC subjects this correlation missing (see Table 2).

### Ability to Switch

The ability to switch in the OCD patients was impaired to change task performance from the detection of rare targets (the CPT) to the very frequent presentation of targets (the Go/NoGo task) (from second to third block). Depending on the moment of reversal probability of the Go and NoGo signals, inflexibility is reflected by the inability to speed responses after changes in the Go signals frequency. Thus, a lower difference in RT between a baseline block and a subsequent block reflects low ability to switch (Figure 1A).

**Figure 2:**
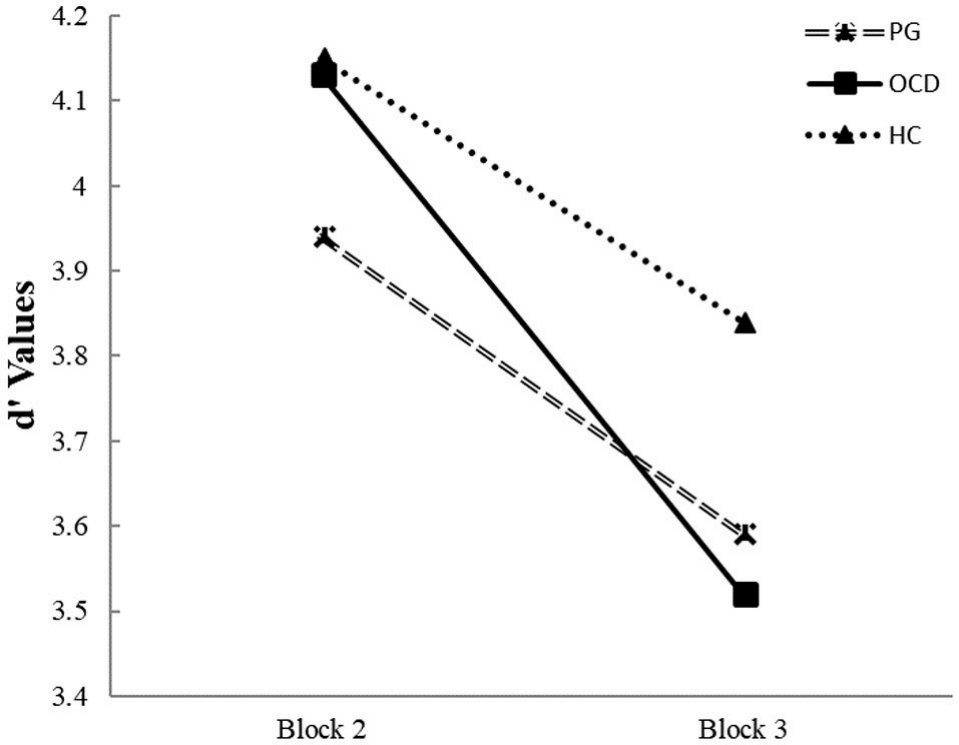
Average perceptual sensitivity (d') in pathological gamblers, obsessive compulsive disorder and healthy control groups for shift costs in changing response from second block to third block (“attenuated response inhibition”). Legend: Average of d' values for the second block (last part of the CPT- rare Go signal) compared to average d' value for the third block (the first part of the Go/NoGo task—frequent Go signals). Different lines connect d' scores in two blocks for each group. In the OCD group, the perceptual sensitivity value decreased significantly more prominent from the detection of rare targets to the frequent presentation of targets from second to third block than in PG and HC groups.

Repeated measurer ANOVA of interaction between group and switch capacity (from second to third block) indicated on main effects of this interaction on d' values [*F*_(2, 298)_ = 6.20, *p* < 0.01]. This interaction indicated that in the second block both OCD and HC groups show greater d' values than the PG group [*t*_(168)_ = 1.71, *p* < 0.05]; *t*_(238)_ = 2.96, *p* < 0.05]. In the second block, there were no differences in the d' value between HC and OCD groups [*t*_(190)_ = 1.05, *p* = NS]. However, in the third block there were no differences between OCD and PG [*t*_(168)_ = 0.53, *p* = NS], but HC participants had greater d' values than both OCD and PG patients [*t*_(190)_ = 3.51, *p* < 0.05; *t*_(238)_ = 3.19, *p* < 0.05]. Further analysis indicated that reduction in the d' values between second to third blocks was greater for the OCD group than in both HC and PG groups [*F*_(2, 298)_ = 6.20, *p* < 0.05)]. No statistically significant difference found between HC and PG groups in level of reduction in the d' values between second to third blocks (Figure 2).

Repeated measurer ANOVA of interaction between group and switch capacity (from second to third block) indicated on main effects of this interaction on C' values [*F*_(1, 298)_ = 1836.4, *p* < 0.01. In all groups *C*-value was greater in the second block compared with third block. Main effect of group [*F*_(2, 298)_ = 4.52, *p* < 0.05] indicated that HC had lower C levels than PG [*t*_(238)_ = 2.73, *p* < 0.05]. No statistically significant difference between HC vs. OCD groups [*t*_(190)_ = 2.668, *p* = 0.08], and no differences between OCD vs. PG patients [*t*_(168)_ = 0.05, *p* = NS].

### Differences Between Groups in d' And C Values

One-way ANOVAs were conducted to explore the effect of group on d' and C indices (Table 2). In the CPT, analysis revealed the main effect of group on both d' [*F*_(2, 298)_ = 10.90, *p* < 0.01] and C levels [*F*_(2, 298)_ = 11.19, *p* < 0.01]. Bonferroni's correction *post-hoc* indicated that PG subjects had lower d' values than HC subjects [*t*_(238)_ = 4.51, *p* < 0.01, Cohen's *d* = 0.61]. And that there was marginal difference in d' levels between OCD vs. PG [*t*_(168)_ = 2.34, *p* = 0.06, Cohen's *d* = 0.06]. No statistically significant difference found between OCD vs. HC [*t*_(190)_ = 1.47, *p* = 0.42]. A contrasting picture was revealed for C values; the PG group had greater C values than the HC group [*t*_(238)_ = 4.72, *p* < 0.01, Cohen's *d* = 0.59]. There was marginal difference between PG and OCD groups [*t*_(168)_ = 2.3, *p* = 0.06, Cohen's *d* = 0.3] and no difference between OCD and HC groups [*t*_(190)_ = 1.57, *p* = 0.35, Cohen's *d* = 0.38].

For Go/NoGo task, there was a main effect of group only on d' value [*F*_(2, 298)_ = 5.59, *p* < 0.01]. Bonferroni's correction *post-hoc* revealed that the HC group had greater d' values than the PG group [*t*_(238)_ = 3.06, *p* < 0.01; Cohen's *d* = 0.41] and the OCD group [*t*_(190)_ = 2.04, *p* = 0.05; Cohen's *d* = 0.47]. There were not differences between OCD and PG patients [*t*_(168)_ = 0.14, *p* = 1, Cohen's *d* = 0.01]. For C values, OCD and PG groups were not differ in C levels [*t*_(168)_ = 1.20, *p* = 0.7, Cohen's *d* = 0.16], HC had marginal lower C values than OCD group [*t*_(190)_ = 2.21, *p* = 0.08, Cohen's *d* = 0.16], and not differ from PG group [*t*_(238)_ = 1.16, *p* = 0.74, Cohen's *d* = 0.164].

As can be seen in Table 1, both d' and C values decreased across switch from low to high frequency of Go signals. The larger number of errors consistently in the high Go frequency condition with the d' finding indicating that the Go/NoGo task appeared to be a more effortful and difficult condition than the CPT for both OCD and PG groups. In all three groups a conservative, risk-averse decision making decreased to a liberal, risk-taking decision making during changing from the detection of rare targets (the CPT) to the very frequent presentation of targets (the Go/NoGo task) from second to third block.

## Discussion

The aims of our research were to compare parameters of the CPT and the Go/NoGo tasks in OCD and PG compared to HC. Although, in some measures, both PG and OCD patients performed worse than HC subjects, specific cognitive performance patterns differentiated between PG and OCD subjects. We assessed the response inhibition of the participants in terms of their RTs, variability of RTs, false alarms, omissions, d' and C indices, and the response switch measure. Our research is one of the first studies to demonstrate that subjects with OCD and PG show distinct patterns of performance on the CPT and Go/NoGo tasks.

### Response Time

Both OCD and PG groups were significantly slower than the HC group. It may be possible that in PG and OCD slowness of RT can be a result of different cognitive mechanisms. In the CPT, slowness of RT was negatively associated with the number of omission errors in PG subjects and positively associated in OCD patients. We can to hypothecate that in PG slowness of RT may be result of inhibition impairments. Cheung et al. (51) suggests that the slowness of the RTs in the Go/NoGo task is a more sensitive measurement of inhibition impairment than the number of false alarms. More specifically, slowness can be a result of inhibition impairment in situations that involve competing voluntary and automatic tendencies and as result is cause a response conflict (52). This conflict is a result of a deficit in the organization of stimulus-response schemata (53). In contrast, in OCD, reduced processing speed may be the primary deficit because it may underlie deficient performance on tests that assess different domains (26, 54). OCD patients are more often significantly slower than are HC individuals in everyday activities such as eating and dressing, and they may perform poorly on time-limited and time-unlimited tasks (55). Alarcón et al. revealed elevation of the mean RT on the Go/NoGo test in OCD patients (55). The slowness seen in OCD patients is independent from psychopathological symptoms (29, 56). Abramovitch et al. (57) suggests that the intrusion of obsessive thoughts overloads the control system in a way that is similar to having numerous open programs on a personal computer, which overloads RAM memory and causes the primary program to operate more slowly. Another reason for understanding response slowness in OCD patients introduce Purcell (58), who identified motor slowness as a possible factor for this phenomena.

### Variability of Response Time

A stable and vigilant level of attention is critical for effective performance in the CPT and Go/NoGo tasks. Both the PG and OCD groups demonstrated less stable performance than the HC group. The variability of RT was significantly higher in both the PG and OCD groups in comparison with the control group. However, this finding had only a small effect size (η^2^ = 0.09). High sensitivity of participant to distraction by external or internal stimuli might cause a slow and variable responses (a fluctuation in processing speed) during task performance. The increased variability of RT is not specific and it has been observed in individuals with high-functioning autism, psychotic spectrum, bipolar disorder with psychosis, traumatic brain injury, early stages of dementia and ADHD (59). Some authors suggested that slow and variable responses may be more related to impulsivity rather than to inattention (60). Our findings are in accordance with the study that did find an increased variability of RT in an OCD sample (61).

### False Alarms

Previously, subjects with impaired inhibition have been found to make more false alarms than HC (61). Both the CPT and Go/NoGo tasks have clear and distinct Go and NoGo stimuli that should result in even perfect performance because of the undemanding discrimination between the two stimuli. We did not find higher number of false alarms during the CPT and Go/NoGo performance in OCD and PG groups as compared to HC. This evidence converges with a recent review that reported a small effect size of commission errors/false alarms (Cohen's *d* = −0.33) for differences between OCD and HC [(26); p. 1168]. A meta-analysis found small-to-medium effect sizes of the number of false alarms across a wide range of mental disorders and concluded that this measure is not specific (62).

### Omission Errors

PG subjects made more omission errors than HC subjects with a moderate effect size. No statistically significant difference found in omission errors between the OCD and HC groups. The number of omission errors is usually considered to be a measure of inattention (63). Inattentive individuals required more time for performance, and the processing of information is delayed. Long RTs is associate with omission errors as omission errors is result of attentional lapses, suggesting that long RTs, the primary cause of response variability (59). In our study, the PG subjects demonstrated impairments on both attention (the CPT) and inhibition (the Go/NoGo task) functions. The presence of ADHD during childhood has been proposed as a possible risk factor for the development of PG in adulthood (64–66). However, the PG patients with a retrospective diagnosis of ADHD did not exhibit more impaired CPT (67) or Go/NoGo (68) performance than the PG without this diagnosis. Although, PG patients showed significantly poorer performance on the CPT and Go/NoGo tasks than did the HC subjects, it cannot to conclude that this deficits is result of the existence of ADHD during childhood.

### Speed–Accuracy Trade-Off

The speed–accuracy trade-off may help to clarify the mechanism of inhibitory impairment. Impulsive participants would seem to prefer greater speed at the cost of accuracy, which indicates a fast response to the NoGo stimuli (69). Thus, we expected that participants with impulsive response type should express a negative correlation between RTs and false alarms. Our results show that only the PG patients showed a significant negative correlation between false alarms and RTs (a fast-errors trade-off) in the Go/NoGo task.

In the CPT, the number of omissions was positively correlated with RTs only in OCD patients. In OCD, an inability to generate the normal “feeling of knowing,” the tendency to overestimate wrong response, create an over-control of mental processes required more detailed processing and more prolonged reaction to a particular stimuli. This excessively high standards for performance accompanied by tendencies for overly critical evaluations of one's behavior (70) as an attempt to prevent a wrong response from occurring reactions. Thus, in OCD the “concern over mistakes” and “doubts about actions” is associated (29). Namely, OCD participants need to have a stringent criterion for certainty that the appropriate response to a particular stimulus is correct before they make it. In this line, slowness of RT was associated positively and significantly with the number of omission errors in the Go/NoGo task only among OCD patients (Table 2). In OCD patients, a slower RT can help to avoid false alarms responses but it might have come at the expense of failing to detect Go signal (omissions).

### Ability to Switch

Task switching involves especially “task management” processes and/or “attenuated response inhibition,” which both require focusing one's attention on relevant information and inhibiting irrelevant signals (46). Difficulties in set-shifting have manifest as rigid approaches to problem solving or difficulties managing dynamic interactions. We found that in transition of performance from the rare Go stimuli to frequent Go stimuli exacerbates set-shifting difficulties on a computerized task only among OCD participants. The OCD subjects continued to use their previous pattern of responses in new situations, and therefore, the difference in RT between the second and third block was significantly lower than in both the PG and HC groups (Figure 1A). The OCD patients expressed a higher switch cost compared to both the PG and HC groups. Our findings in accordance with a recent review that reported a small medium effect size of cognitive flexibility (Cohen's *d* = – 0.517) for differences between OCD and HC [(26) p. 1168]. This finding aligns with an earlier suggestion that the cognitive inflexibility of OCD patients is expressed by ineffective adaptation to a changing environment (71). This phenomenon was reported also in unaffected relatives of OCD subjects (71). In addition, OCD patients demonstrated impairment of implicitly learning probabilistic associations between events (72). Furthermore, flexibility is required when responses that previously associated with positive or negative outcomes should be switched to new opposite consequences. Reduced sensitivity to the outcome of actions can promote decreased flexibility in a dynamic environment due to a deficits in the action feedback that cause diminished need to update action goals (73). Thus, at present it remains unclear which particular mechanism is responsible for the cognitive inflexibility in patients with OCD.

In addition, in the CPT, the frequency of NoGo stimuli was high and promotes a tendency to be conservative toward missing rare Go stimuli. During a switch from this situation to the Go/NoGo task with rare NoGo events that require a rapid response to frequent Go stimuli, the OCD subjects continue to perform in the same style (Figure 2). During a switch OCD patients show the significantly higher decrement of d' value than the HC and PG groups (Figure 2). In contrast, the performance of the PG group was equal to the HC group. This result was concordant with the finding of a previous experiment (74) that used another switch paradigm, the Deterministic Reversal Learning Task (DRLT), in a similar population. In the DRLT, the stimulus is entirely predictive of the outcome (reward or punishment) rather than probabilistic as in our paradigm. In the DRLT, perseverative responding is associated with an elevation in error rate after reversal. Janssen et al. (74) reported no differences in performance between PG and HC groups on this task. Vanes et al. (75), using another task, i.e., the Contingency Learning Task (CLT), also failed to detect differences in cognitive flexibility between PG and HC subjects. The same results were found in a study that used the switching task (76).

### Differences in Performance Between PG and OCD

In the CPT, scores above three in all three groups suggests that the stimulus is easily discriminated from the background noise (47). PG patients exhibited lower d' values compared to HC group. The PG group also exhibited greater C values than the HC group. There were no differences in d' and C values between the PG and OCD groups. PG subjects used a more conservative response criterion than the HC group. A conservative response criterion is a response to a signal only if the examinee is very sure, indicating a high frequency of correct rejection and a low frequency of false alarms. This finding was in contrast to our expectation for using a liberal approach in the PG group during the CPT performance. The differences in C values between the PG and OCD groups did not found. These results are consistent with previous studies that have been proposed overactive error monitoring as a phenotype for OCD (77). It was expected that in the OCD subjects, an accurate but slower performance could be the result of a cognitive strategy that prefers control rather than automatic responses (70). We anticipated that effortful processing in the Go/NoGo task should produce decreasing of the d' value relatively to the CPT condition. As expected, in the Go/NoGo effortful condition, HC subjects had greater d' values than both PG and OCD patients. No statistically significant difference found between the OCD and PG groups. The lower accuracy and faster responding in the Go/NoGo task as compared with the CPT seems to be due to a change in the both decision criteria and a faster processing (Table 2) (78). In contrast to our expectation for an impulsive over-responding (liberal) approach in the PG group, they respond in the same approach as the HC group. These results highlight that PG in effortful condition not produce more random, haphazard errors (increased false alarms rate) (41). When Go stimuli are presented at a relatively frequent rate, individuals become alerted and prepared for immediate response. As the interval between Go stimuli shortens, there tends to be a general elevation in tonic arousal, which elevates preparatory alertness and reduces the RT. In general, in the effortful situation, the subjects with the faster RTs showed a more evident over-responding criteria, which could optimize accurate detections of the high frequency of Go signals.

### Strengths and Weaknesses of the Present Study

Major strength of our research is the inclusion of medication-free population. Both OCD and PG are complex disorders with many symptoms and variants that have been associated with involvement of variable response inhibition impairments. It would be simplistic to propose that impaired response inhibition could explain exclusively the entire cognitive dysfunction detected in these disorders. Furthermore, we did not assess the presence of personality disorders in our OCD and PG participants, which may have had an impact on their neurocognitive performance (79). Therefore, it is impossible to assume that the response criterion that the participants adopted in the laboratory situation are the same as that they employed in real-world actions.

## Conclusions

Our findings have important implications for the understanding of differences in cognitive performance patterns between OCD and the PG and its assessment.

PG subjects showed the poor response inhibition and under-controlled behavioral style, as reflected by the C index in the CPT and a negative correlation between false alarms and RT (a fast-error trade-off) in the Go/NoGo task. In contrast, the OCD group display over-controlled style of performance such a positive correlation between omission errors and RT in the CPT and the impaired ability to switch when they were required to change responses depending to reversal probability of the Go signal. The question regarding association between impaired set-shifting in OCD and possible increased perseveration was beyond aims of the current study. Our findings have clinical implications that the measures of the CPT and the Go/NoGo task linked with each symptom dimension, hypothesized to underlie the development and maintenance of the symptoms, can be targeted in treatment. The noradrenaline deficit associated with insufficient inhibition, but the diminished level of serotonin associated with impaired the probabilistic learning and the reduction of flexibility (80). Future studies would expand the different theories of impulsivity and compulsivity; and explore the specific interaction between these two distinct concepts in terms of interruptive, interference, and waiting inhibitions can help delineate the contribution of specific techniques for the development of personalized treatments of OCD and PG based on specific individual profiles of performance (81). Future studies could be designed to assess the association between performance measures of the response inhibition tasks and self-reported measures of impulsivity in the PG and OCD samples. Also important t to use event-related brain potentials for assessment the brain correlates of response inhibition measures, and explore the Neurocognitive mechanisms of the CPT and the Go/NoGo performance differences between PG and OCD patients.

## Author Contributions

All authors listed have made a substantial, direct and intellectual contribution to the work, and approved it for publication.

### Conflict of Interest Statement

SK and PD are consultants at Animascan which produces neurocognitive test batteries. The remaining authors declare that the research was conducted in the absence of any commercial or financial relationships that could be construed as a potential conflict of interest.
